# Supplementary figures and images for: Ratiometric GPCR signaling enables directional sensing in yeast
Source: PLoS Biol. 2019 Oct 17;17(10):e3000484. doi: 10.1371/journal.pbio.3000484 (PMC6818790; doi:10.1371/journal.pbio.3000484)

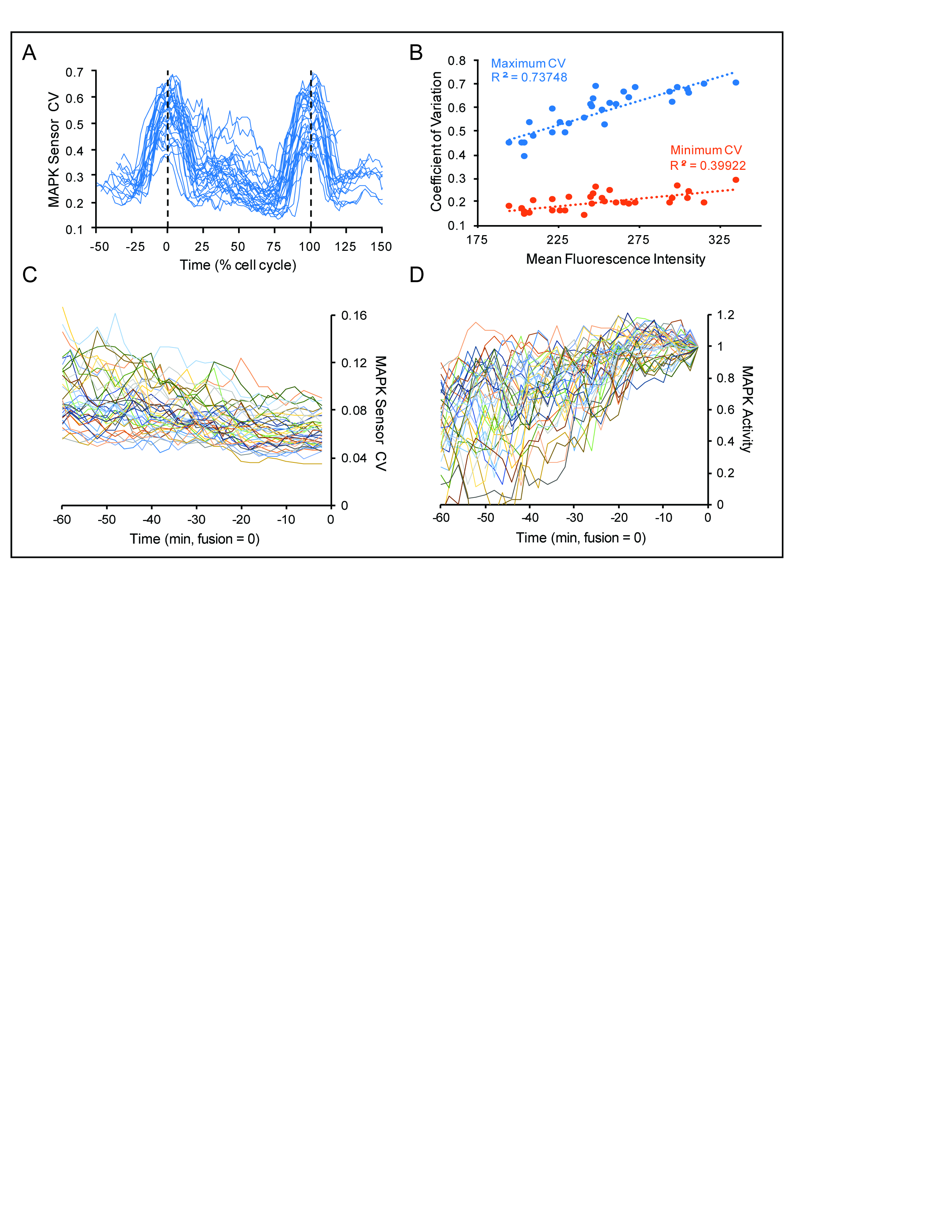

Supplement: S1 Fig — Cells harboring Ste71–33–NLS–NLS–mCherry were imaged for 150 min with 2-min resolution. (A) CV of Ste71–33–NLS–NLS–mCherry, measured from maximum projection images in an ROI encompassing the full cell. Time was normalized to “% cell cycle,” with the first cytokinesis for each cell aligned at 0, and the second cytokinesis aligned at 100. (B) Maximum (blue) and minimum (orange) CV versus mean fluorescence intensity for each cell in (A). Mean fluorescence intensity was measured in the same ROI as the CV and averaged across all time points for each cell. (C) Ste71–33–NLS–NLS–mCherry CV measured as in (A) for mating cells. For each cell, fusion was designated as 0 min, and the timeline extends back 60 min. (D) MAPK activity metric plotted for the same cells shown in (C). For each cell, the CV at time point before fusion was normalized to 1. Normalized CV values were then subtracted from 2 to generate a MAPK activity metric that rises to a value of 1 just before fusion. Strains: DLY22259 (A–D). CV, coefficient of variation; MAPK, mitogen-activated protein kinase; NLS, nuclear localization sequence; ROI, region of interest; Ste, sterile. (TIF) [file pbio.3000484.s001.tif]

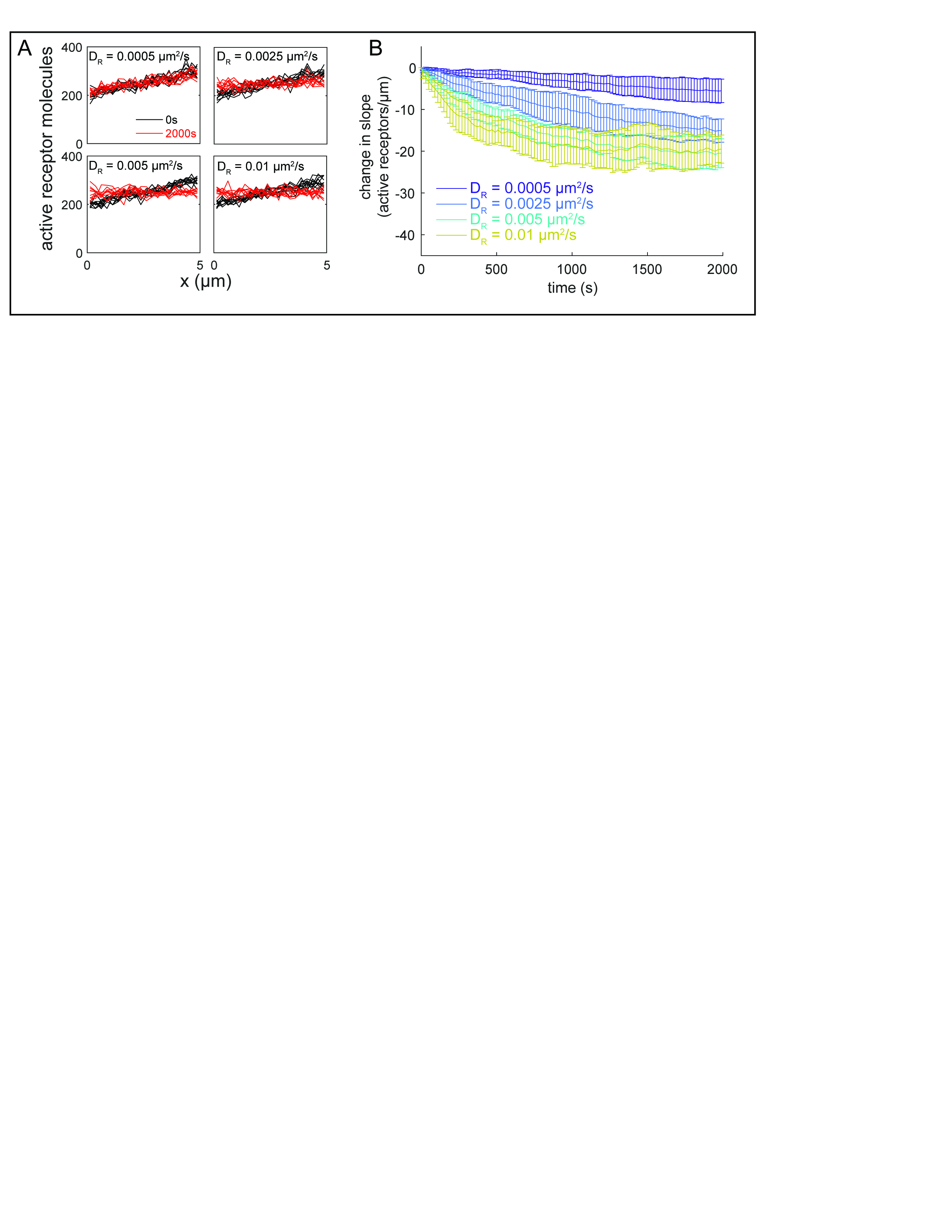

Supplement: S2 Fig — (A) Snapshots of the active receptor gradient at t = 0 (black) and 2,000 s (red) for different values of the diffusion coefficient. Each curve represents a histogram with 250 nm bins derived from a single simulation. (B) Decay of the active receptor gradient as measured by the slopes of linear regressions fitted to the data in (A). The results show the mean of 10 realizations ± 1 SD for the four diffusion coefficients tested. Code and key data are available at https://github.com/mikepab/ratiometric-gpcr-particle-sims. (TIF) [file pbio.3000484.s002.tif]

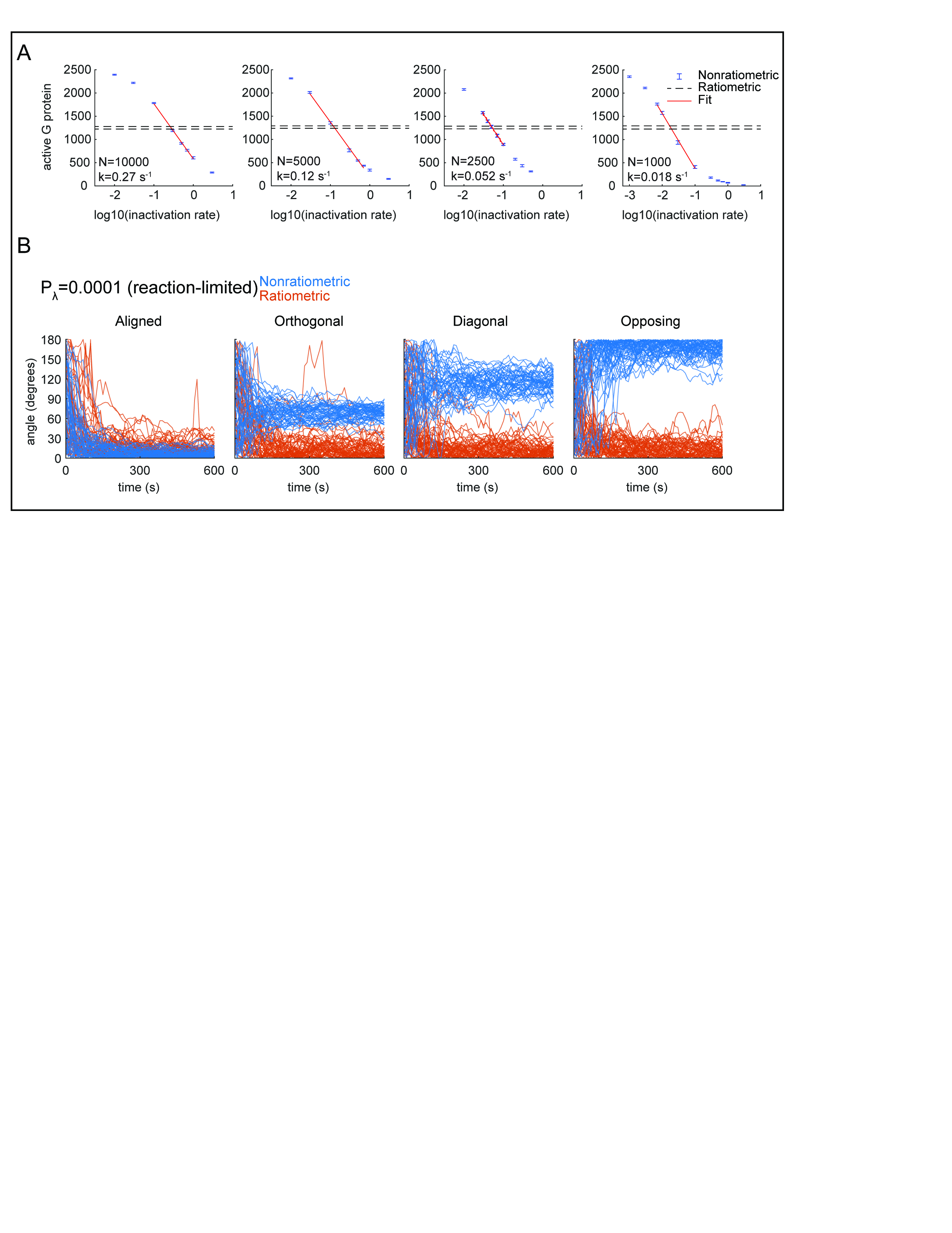

Supplement: S3 Fig — (A) G-protein inactivation rate constant calibration, relating the nonratiometric and ratiometric models. The results shown are for the mean of 10 simulations for each condition, and the error bars represent ± 1 SD. Changing the number of receptor molecules (N) requires recalibration of the inactivation rate in the nonratiometric model. (B) Effect of decreasing the reaction rates to a reaction-limited regime (Pλ = 0.0001 per time step). The corresponding nonratiometric G-protein inactivation rate was k = 0.0031 s−1. The results shown are for 50 realizations of each model. Although it now takes longer for simulations to reach steady state, once at steady state, the G-protein distributions are similar to those in the diffusion-limited scenario. Code and key data are available at https://github.com/mikepab/ratiometric-gpcr-particle-sims. (TIF) [file pbio.3000484.s003.tif]

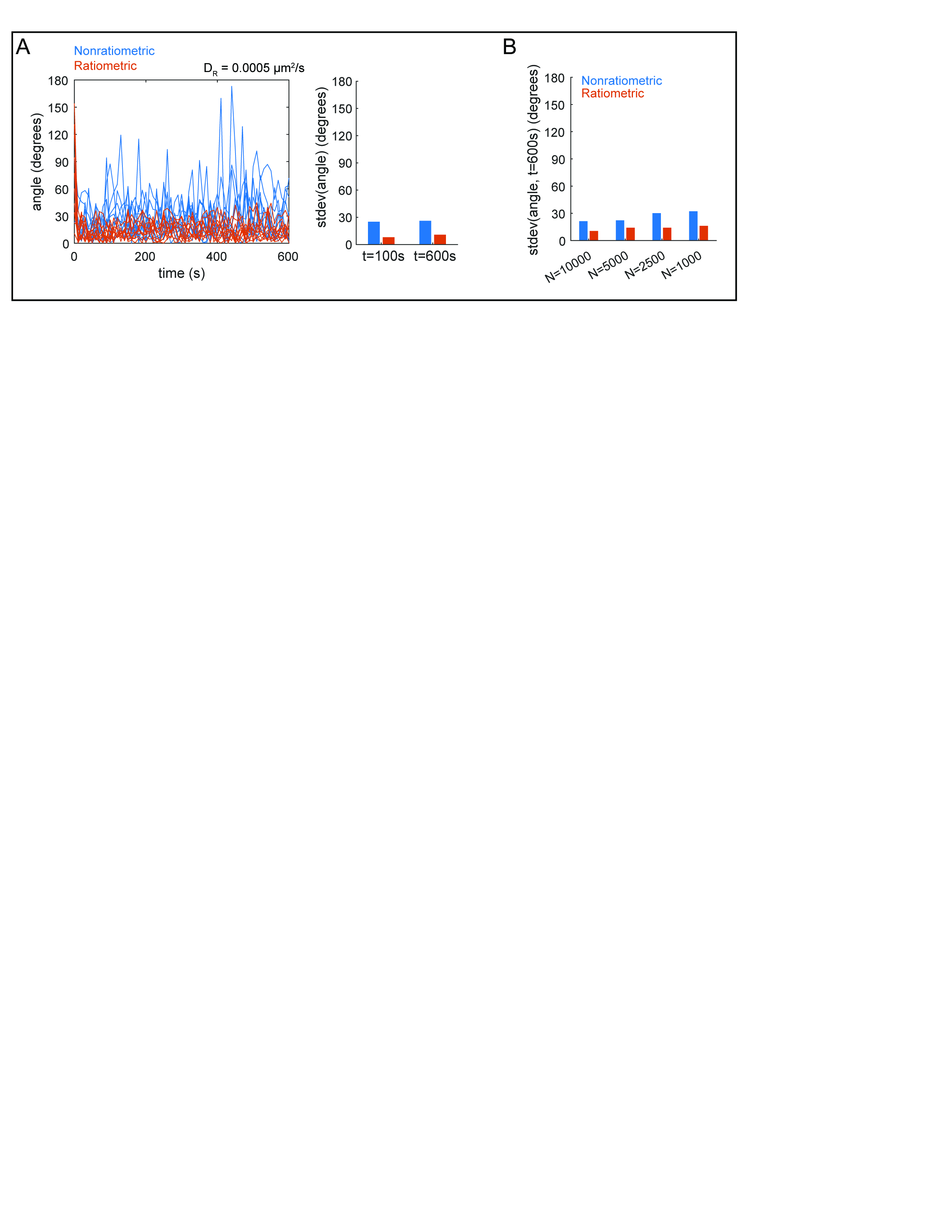

Supplement: S4 Fig — (A) Accuracy of G-protein activity gradients for the nonratiometric (blue) and ratiometric (orange) models with uniform receptor density, as in Fig 8E but allowing receptor diffusion at D = 0.0005 μm2/s. Left: illustrative simulation with measurements every 10 seconds. Right: Variability in orientation angle from 10 simulations of each model, at t = 100 s and 600 s snapshots (SD). (B) Effect of decreasing receptor abundance. Variability in orientation angle from 50 simulations of each condition. Code and key data are available at https://github.com/mikepab/ratiometric-gpcr-particle-sims. (TIF) [file pbio.3000484.s004.tif]

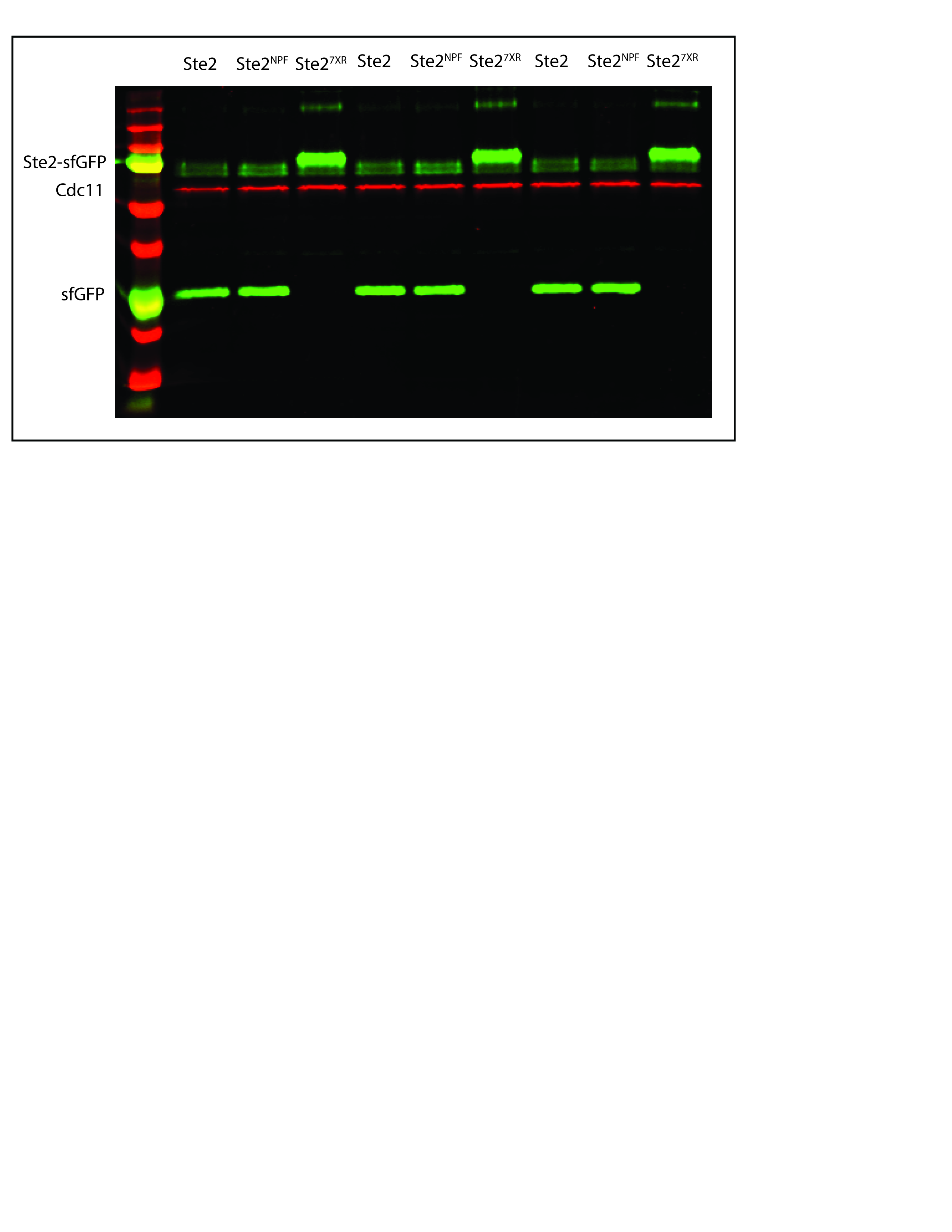

Supplement: S5 Fig — Uncropped western blot used to generate Fig 7C. α-GFP antibodies (green) label two bands—full-length Ste2–sfGFP and vacuolar sfGFP (note absence of vacuole signal for Ste27XR-GPAAD). α-Cdc11 antibodies (red) label Cdc11 (loading control). Cdc, cell division control; GFP, green fluorescent protein; sf, superfolder; Ste, sterile. (TIF) [file pbio.3000484.s005.tif]
