# Supplementary material for: Ratiometric GPCR signaling enables directional sensing in yeast
Source: PLoS Biol. 2019 Oct 17;17(10):e3000484. doi: 10.1371/journal.pbio.3000484 (PMC6818790; doi:10.1371/journal.pbio.3000484)
Supplement: S1 Text — (PDF) [file pbio.3000484.s010.pdf]

Supplementary material: Comparison of ratiometric and nonratiometric models

1 Notation and assumptions

Our model is one-dimensional, and considers the concentration of receptors and G proteins at each point x along a fixed axis aligned with the pheromone concentration gradient. Whereas concentrations of active and inactive proteins will vary with x, we assume uniform concentrations of total receptor and G protein. We use the following nomenclature:

L	diameter of the cell
x	position along the axis, $x \in [0, L]$
$C(x)$	pheromone concentration gradient (extracellular)
K_D	dissociation constant for pheromone-receptor binding
$R(x)$	concentration of inactive receptor
$R^*(x)$	concentration of active receptor
R_{tot}	total receptor concentration, $R_{tot} = R + R^*$
$G(x)$	inactive G protein concentration
$G^*(x)$	active G protein concentration
G_{tot}	total G protein concentration, $G_{tot} = G + G^*$
D_R	diffusion constant for receptors
D_G	diffusion constant for G protein
k_a	G protein activation rate constant
k_i	G protein inactivation rate constant for ratiometric model
k_i'	G protein inactivation rate constant for nonratiometric model
$\beta = k_i/k_a$	ratio of the rate constants
h	steepness parameter for modeled R^* gradient

To compare the ratiometric and nonratiometric models, we assume that $k_i' = 0.5 k_i R_{tot}$. We assume that diffusion of the receptor is negligible: $D_R = 0$.

We assume that all concentrations are in steady state, and thus independent of time.

For an arbitrary external pheromone concentration $C(x)$, the steady-state concentration of active receptor would be:

$$R^*(x) = \frac{C(x)}{K_D + C(x)} R_{tot}$$

For the yeast system, the steady-state assumption for active receptors does not apply because pheromone binding and unbinding are slow. In what follows, we consider an arbitrary receptor activity gradient $R^*(x)$, with $R(x) = R_{tot} - R^*(x)$; for this reason, $C(x)$ does not appear explicitly in the model.

2 Ratiometric model

Given $R^*(x)$ and $R(x)$, the steady-state concentrations of active and inactive G protein satisfy

$$k_i R G^* = k_a R^* G \tag{2.1}$$

for $x \in [0, L]$. Since $G(x) = G_{tot} - G^*(x)$, we may solve for $G^*(x)$ to obtain

$$\frac{G^*(x)}{G_{tot}} = \frac{k_a R^*(x)}{k_i R(x) + k_a R^*(x)} = \frac{R^*(x)}{\beta(R_{tot} - R^*(x)) + R^*(x)} \tag{2.2}$$

since $k_i = \beta k_a$.

3 Nonratiometric model

For the nonratiometric model, we model the inactivation of G protein by $k_i' G^*$ rather than $k_i R G^*$. Therefore, the steady-state concentrations of active and inactive G protein satisfy

$$k_i' G^* = k_a R^* G \tag{3.3}$$

for $x \in [0, L]$. Substituting $G(x) = G_{tot} - G^*(x)$ and solving for G^*, we obtain

$$\frac{G^*(x)}{G_{tot}} = \frac{k_a R^*(x)}{k_i' + k_a R^*(x)} = \frac{R^*(x)}{0.5\beta R_{tot} + R^*(x)}, \tag{3.4}$$

where we have used the assumption that $k_i' = 0.5 k_i R_{tot} = 0.5 \beta k_a R_{tot}$.

4 Small diffusion approximation

In both of these models, we have neglected diffusion of the G protein. The diffusion constant D_G for the G protein has not been measured, but can be estimated to be $D_G < 0.02\,\mu m^2/s$ based on measurements for Cdc42, another peripheral membrane protein in budding yeast [1]. The ratio L^2/D_G may be regarded as the time-scale for a G protein particle to diffuse across the cell; with $L = 5\,\mu m$, this time scale is > 20 minutes. In comparison, the timescale for the activation reaction ($1/(k_a R_{tot})$) is ≤ 1 minute [2]. Therefore, it is reasonable to neglect diffusion in the model. Starting from a reaction diffusion system

$$\partial_t G^* = D_G \partial_x^2 G^* - k_i R G^* + k_a R^* G = 0,$$
$$\partial_t G = D_G \partial_x^2 G + k_i R G^* - k_a R^* G = 0,$$

for the steady state G protein concentrations, one obtains the ratiometric model (2.1) by setting $D_G = 0$. Similarly, the nonratiometric model (3.3) follows from setting $D_G = 0$ in the system

$$\partial_t G^* = D_G \partial_x^2 G^* - k_i' G^* + k_a R^* G = 0,$$
$$\partial_t G = D_G \partial_x^2 G + k_i' G^* - k_a R^* G = 0.$$

5 Comparison of the two models

Given the active G protein gradient G^*, let us define the quantity

$$\Delta G^* = G^*(L) - G^*(0),$$

which is the difference in protein concentration across the length of the cell. To quantitatively compare the ratiometric and nonratiometric models, we define the signal ratio

$$SR = \frac{\Delta G^*(\text{ratiometric})}{\Delta G^*(\text{nonratiometric})}. \tag{5.5}$$

We now suppose that the active receptor gradient $R^*(x)$ is an increasing linear function on the interval $[0, L]$, with the value $R^*(L/2) = 0.5R_{tot}$ at the midpoint. Thus, R^* takes the form

$$R^*(x) = R_{tot}\left(\frac{1}{2} + h(\frac{x}{L} - \frac{1}{2})\right) \tag{5.6}$$

where $h \in [0, 1]$ is a "steepness" parameter. If $h = 0$, then $R^*(x) = R_{tot}/2$ is constant. If $h = 1$, then $R^*(0) = 0$ and $R^*(L) = R_{tot}$. In all cases $h \in (0, 1]$; small $h \approx 0$ corresponds to a shallow pheromone gradient, while large $h \approx 1$ corresponds to a very steep pheromone gradient.

The ratiometric model (2.2) may now be written as

$$\frac{G^*(x)}{G_{tot}} = \frac{R^*(x)}{\beta R^*(L/2) + R^*(x) + \beta(R^*(L/2) - R^*(x))}, \tag{5.7}$$

while the nonratiometric model (3.4) is

$$\frac{G^*(x)}{G_{tot}} = \frac{R^*(x)}{0.5\beta R_{tot} + R^*(x)} = \frac{R^*(x)}{\beta R^*(L/2) + R^*(x)}. \tag{5.8}$$

At $x = L/2$, the fraction of active G protein in the two models (formulas (5.7) and (5.8)) is the same. However, the expression $\beta(R^*(L/2) - R^*(x))$ in the denominator of the ratiometric expression (5.7) is an increasing function of x (assuming $h > 0$). As a result, we always have

$$\Delta G^*(\text{ratiometric}) > \Delta G^*(\text{nonratiometric})$$

as long as $R^*(x)$ is not flat (i.e. $h \neq 0$). Therefore, the signal ratio SR is larger than 1 if $h \neq 0$, which indicates that the ratiometric mechanism enhances the signal, compared to the nonratiometric

system. The formula for the signal ratio is:

$$SR = \frac{\Delta G^*(\text{ratiometric})}{\Delta G^*(\text{nonratiometric})} \tag{5.9}$$

$$= \frac{\frac{R^*(L)}{\beta R^*(L/2)+R^*(L)+\beta(R^*(L/2)-R^*(L))} - \frac{R^*(0)}{\beta R^*(L/2)+R^*(0)+\beta(R^*(L/2)-R^*(0))}}{\frac{R^*(L)}{\beta R^*(L/2)+R^*(L)} - \frac{R^*(0)}{\beta R^*(L/2)+R^*(0)}}. \tag{5.10}$$

with R^* given by (5.6). This simplifies to

$$SR = \frac{2(\beta R^*(L/2) + R^*(L))(\beta R^*(L/2) + R^*(0))}{(\beta(R_{tot} - R^*(L)) + R^*(L))(\beta(R_{tot} - R^*(0)) + R^*(0))}. \tag{5.11}$$

In Figure 8B, we plot the linear receptor gradient R^* (5.6) along with the normalized active G protein gradient $G^*(x)/G_{tot}$ for both the ratiometric (5.7) and nonratiometric models (5.8). In Figure 8C, we plot the signal ratio (5.11) as a function of β and the steepness parameter h. As explained above, we observe that $SR > 1$ for all β. For shallow receptor gradients (h small), the signal ratio SR is approximately 2, meaning that the active G protein gradient is approximately twice as large in the ratiometric model compared to the nonratiometric model. For $h = 1$, corresponding to the steepest receptor gradient, the signal ratio simplifies to

$$SR = 1 + \frac{\beta}{2}.$$

In particular, the signal ratio is an increasing function of $\beta = k_i/k_a$. Of course, as β increases, the levels of G^* decrease, so that the overall signal in both models is reduced.

For both models, the function $G^*(x)$ is increasing with x (assuming that h and β are not zero), following the gradient in the receptor concentration. By computing the second derivative of $G^*(x)$, one finds that $\frac{d^2}{dx^2}G^*(x)$ is always negative for the nonratiometric model (3.4), which means that $G^*(x)$ is a concave function. On the other hand, for the ratiometric model (2.2), $\frac{d^2}{dx^2}G^*(x)$ is positive when $\beta > 1$, indicating that $G^*(x)$ is convex in this case. This implies that with the ratiometric model and $\beta > 1$, the active G protein concentration is more concentrated near the high end than it would be for the nonratiometric model, even if the end-to-end differences ΔG^* were the same, as illustrated in Figure 8D.

References

1. Sartorel E, Unlu C, Jose M, Massoni-Laporte A, Meca J, Sibarita JB, et al. Phosphatidylserine and GTPase activation control Cdc42 nanoclustering to counter dissipative diffusion. Mol Biol Cell. 2018;29(11):1299-310. Epub 2018/04/19. doi: 10.1091/mbc.E18-01-0051. PubMed PMID: 29668348; PubMed Central PMCID: PMCPMC5994902.

2. Bush A, Vasen G, Constantinou A, Dunayevich P, Patop IL, Blaustein M, et al. Yeast GPCR signaling reflects the fraction of occupied receptors, not the number. Mol Syst Biol. 2016;12(12). doi: 10.15252/msb.20166910. PubMed PMID: 28034910.
