# Supplementary material for: Ratiometric GPCR signaling enables directional sensing in yeast
Source: PLoS Biol. 2019 Oct 17;17(10):e3000484. doi: 10.1371/journal.pbio.3000484 (PMC6818790; doi:10.1371/journal.pbio.3000484)
Supplement: Attachment — Submitted filename: Response to reviewers.docx [file pbio.3000484.s012.docx]

*We thank the reviewers for their thorough and constructive comments. In the response below, specific reviewer points are followed by our responses in italics.*

---------------

Reviewer #1 (Jeffrey Segall)

---------------

One aspect that it would be interesting for the authors to provide more details on from their current data (unless I missed it) is whether there are differences in Bem1/spa2 cluster kinetics of a vs alpha cells.

***Response:*** *we have not seen any mating-type-specific differences in the behavior of polarity markers during mating. We have added a panel (new Fig. 2D and lines 176-7) comparing the durations of the indecisive and committed phases in a and alpha cells.*

Also, for the STE5 construct, if the authors did made the measurement - did the construct colocalize with the spa2/Bem1 probes?

***Response:*** *our STE5 construct was not fluorescent so we cannot answer this.*

Editorial point:

Reference on line 467 should be 37, not Bush et al 2016.

***Response:*** *thank you—now fixed.*

---------------

Reviewer #2

---------------

1.  Regarding the “Timing of commitment” section (Fig 2 and lines 163-187):  Although the “simplest hypothesis” described is reasonable, there is a good chance it is considerably more complex and interesting, in a way that might be worth discussing. In particular, the direction of pheromone secretion might be as relevant as the pheromone expression level. That is, for the first-born cell to reach the committed phase, it might require the second-born cell to not only reach G1 but also to start tentatively showing directional pheromone secretion. This relates to the old “courtship” idea of Jackson and Hartwell, in which a “conversation” between the two mating partners allows each to know that the other is showing a reciprocal commitment, so that cells in a mixture can “pair up” efficiently. How this works is a fascinating and unsolved problem (which the current study might be on the cusp of addressing); it might involve detection by each cell of an increase in pheromone

concentration in the direction in which it is polarizing.

To give a concrete example, I wonder what would happen if the second-born cell was unable to follow a gradient, such as a cdc24-m mutant. Would the first-born cell still be equally likely to reach the committed phase once its mutant partner reached G1, or would it do so only infrequently (i.e., when it happened to lie in the default polarization direction of its partner), because it requires a further indication that the partner is reciprocating? Similarly, in the existing data (i.e., mating mixtures filmed by the authors), there should be instances in which there is more than one potential partner for a given cell, and so it should be possible to examine the polarization behavior of the potential partner that is *not* chosen. Does it ever reach the committed phase? I suspect not. In the current Video 1, there seems one such example slightly below and right of center, and another at the very bottom-right of the field.

***Response:*** *we completely agree with this insightful discussion: the issues raised here are fascinating to address, and we are pursuing experiments to that end. However, the issues are complex and deserve a thorough treatment of their own, so we plan to leave them for the next paper.*

2.  Regarding the MAPK activity sensor:

(a) The authors describe a variation in the basal distribution with cell cycle position (Fig 3A), but do not provide any explanation. It seems likely that the sensor is being phosphorylated by CDK activity, which is not surprising given the sequences of the phosphorylation sites and the fact that the NLS is derived from a CDK-regulated target (Swi6). It seems worth stating this directly rather than leaving it seeming mysterious.

(b) As the authors acknowledge, it is not clear if the plateau in the MAPK sensor reflects a real plateau in MAPK activity or simply saturation of the sensor. In principle this could be resolved by comparing in parallel the dose response profiles for the sensor and for conventional MAPK phosphorylation via a western blot (e.g., to see if their EC50s are similar, or if the sensor plateaus before MAPK phosphorylation does). It is unfortunate that this was never done in the original sensor paper. It is not critical here, but if the authors wanted to draw a stronger conclusion about the observed plateau then this approach could provide a straightforward way to resolve the ambiguity.

***Response:*** *agreed. We have added a comment to the effect that the sensor may be somewhat responsive to CDK activity (lines 208-11). We have not addressed sensor saturation as it would not affect any of our conclusions in this paper.*

3.  Lines 350-352 describes the elevated protein levels for the Ste2-7XR-GPAAD mutant and ends with a statement that these cells were “more sensitive to pheromone”.  It wasn’t clear to me what point is being made here. As written, it seems to imply that the increased sensitivity is an obvious consequence of the increased protein level. But this is contrary to the ratiometric model, which explains why sensitivity does NOT increase in proportion to receptor number. Presumably the very slight increase in sensitivity observed does not match that predicted for a 4-5x increase in receptor (which, without the ratiometric mechanism, should cause cells to behave as though they are detecting 4-5x more pheromone). And presumably the increase is much less than would be observed in the hsRGS4 background, where the ratiometric mechanism is absent. So, maybe the authors should clarify if they think the “more sensitive” response violates the ratiometric model or is instead a mild-but-measurable level that perhaps indicates that the ratiometric mechanism is not perfect (or not able to function at all stimulus levels).

***Response:*** *we agree that the small increase in pheromone sensitivity is likely due to imperfect ratiometric sensing, not a violation of ratiometric sensing. We have added a comment to that effect. The reviewer is correct that this is much less than what is observed in the hsRGS4 background, and we now include that data in the new Fig. 7E (lines 362-365) to make exactly that point.*

4.  Lines 488-498 discusses some comparisons to other systems. It might be interesting to compare/contrast the results of ratiometric sensing with the “LEGI” (Local Excitation Global Inhibition) models prevalent in other chemotaxis models. The ratiometric mechanism provides an interesting counterpart because the inhibition is not “global” but instead is localized in a pattern that is reciprocal to the excitation (perhaps “LERI”, for Local Excitation Reciprocal Inhibition?). This might be an even more effective mechanism of gradient amplification than LEGI.

***Response:*** *this is an interesting comment, but we are unsure how best to compare LEGI (which involves differential induction kinetics and diffusivity of an activator and an inhibitor) with ratiometric sensing (in which an “inhibitor”, the unbound receptor, is converted to an “activator”, the ligand-bound receptor, with matched kinetics and diffusivity).*

5. Lines 499-510:  I am a bit skeptical of this suggested rationale for the role of receptor endocytosis. First, pheromone exposure stimulates the rate of receptor endocytosis. This seems the opposite of the expectation from the author’s proposal – i.e., if the main point was to clear receptors prior to mating type switching, it would make sense to rapidly clear receptors when cells were not responding to pheromone, and stop clearing them when cells were arrested in G1 and in the process of mating. Second, endocytosis also helps polarize the receptors as cells build the mating projection and grow toward the partner. This seems likely to hint at the real physiological utility. Perhaps receptor polarization helps keep the response directionally persistent, or helps contribute to the “committed” phase that the authors report here. The experiments here with the non-endocytosed receptor (Ste2-7KR) only addressed the initial directional bias and did not comment on whether the later “committed” phase was altered, but older studies reported that endocytosis-defective receptors caused mating projections to be broadened (Konopka 1988). It also seems possible that rapid endocytosis and polarization of receptors during the middle-later stages could help place a demand for increasingly focused secretion of pheromone from a partner cell, which could help cells determine if a partner is mutually reciprocating (see point #1). So, perhaps it is worth considering and discussing some additional, alternative physiological rationales.

***Response:*** *these are insightful comments and we agree that roles for receptor endocytosis are likely much more extensive than just mating-type switching. We have removed that speculation from the Discussion.*

---------------

Reviewer #3

---------------

1.	A model is presented that hypothesizes that cells synchronize their commitment to occur in G1 by increasing the expression of pheromone in G1.  The experiment to establish this is to use the MFalpha1 promoter to drive expression of intracellular GFP.  The authors show that there is a saw-toothed temporal pattern in GFP, decreasing by about 20-30% during S/G2/M.  Although this is consistent with G1 specific expression, it is a difficult experiment to interpret, given that the volume of the cell is changing in a non-linear manner.  There is no control demonstrating what steady state expression of an intracellular GFP actually looks like, so it is hard to know whether it reflects cell cycle regulated expression, and dilution, or some other effect.  Moreover, the magnitude of the effect is relatively small, which decreases the confidence that this is an important effect. Most of the difficulty stems from examining accumulation of a stable protein.  This point needs to

supported by a more direct examination of the mRNA.   I note that there is no data at SGD on the cell cycle regulation of this promoter.

***Response:*** *the reviewer is correct. We have now added a control reporter driven from a constitutive promoter: as expected, this reporter displays a steady (not sawtooth) signal. We have also used modeling (as suggested by Reviewer #4) to extrapolate what difference in synthesis rate between G1 and S/G2/M would be required to yield the observed sawtooth response. Because the stability of the reporter dampens the signal oscillation, the model (using measured GFP maturation and dilution rates) estimates that the true synthesis rate varies about 5-fold through the cell cycle (new Fig. 2F,G and lines 182-189).*

2.	All of the modeling seems to be based on cells responding to stable preformed pheromone gradients.  However, the experiments are typically based on cells of opposite mating type being put in close proximity at t=0, and there is no consideration in the modeling for a temporal component effecting the response as the pheromone starts to diffuse between the cells.  This may not affect the modeling significantly, but this needs to be addressed.

*Response: the reviewer is correct. We have assumed steady gradients in the modeling as a simplest-case scenario, but the complexity and time-varying nature of real-life gradients will be important to understand in order to fully appreciate cellular partner choice. We are working on these issues, but as they are beyond the scope of this paper we simply added a clarification to the text (line 295-297).*

3.	It has recently been shown that there are changes in the localization of components of the polarization apparatus late in mating, just prior to cell fusion, and dependent on cell fusion proteins.  It is unclear what role the cell fusion proteins play in the commitment phase.  While new experiments are likely to be beyond the scope of the paper, the role of cell fusion proteins late in the pathway should be mentioned and discussed in the introduction and discussion.

*Response: we agree that cell fusion proteins may affect polarity protein behavior in the committed phase, and that such effects are beyond the scope of this paper.*

---------------

Reviewer #4

---------------

In Fig. 2D, the signal for ‘pheromone production’ goes from ~0.8 to ~1, about a 20% fluctuation through the cell cycle. This is not much and probably an understatement of the reality, The authors could either used a destabilized fluorescent protein or make a model to estimate protein synthesis and dilution through the cell cycle for individual cells to get a better estimate of that the pheromone production actually is.

*Response: we thank the reviewer for this suggestion. We have made a model to get a better estimate what difference in synthesis rate between G1 and S/G2/M would be required to yield the observed sawtooth response. Because the stability of the reporter dampens the signal oscillation, the model (using measured GFP maturation and dilution rates) estimates that the true synthesis rate varies about 5-fold through the cell cycle (new Fig. 2F,G and lines 182-189).*

In Fig 3A the authors state and show images of fluctuations of the MAPK sensor through the cell cycle. It would be nice if this were quantified and if we knew of this was due to Cdk activity (which can target similar sites) or due to fluctuations in the basal MAPK activity. Obviously, this is not a paper about the sensor, but in the case of these sensors, the more we know about specificity the better. If the fluctuation in the cell cycle is due to Cdk, that does not affect the author’s conclusions about the sensor dynamics in pheromone (where there is no Cdk activity). Would an analog sensitive Fus3 be useful here?

In Figure 6H, the authors should quantify the images as they have for other signals to show the colocalization here of Bem1 and Ste2.

*Response: The fluctuations were quantified in Fig. S1. We suspect (as also suggested by reviewer 2) that the phosphorylation of the sensor during the cell cycle is due to CDK activity, but we have not tested that experimentally. We have added a comment on this (line 208-11).*
